# Mental health disorders and the risk of abuse of prescribed opioids

**DOI:** 10.1192/j.eurpsy.2025.245

**Published:** 2025-08-26

**Authors:** G. Dom

**Affiliations:** University of Antwerp, Antwerp, Belgium

## Abstract

**Abstract:**

Chronic (non-cancer) pain is a rising problem within Western population. Importantly, people with mental illness have disproportionally high levels of (chronic) pain. Prescription opioid medications are frequently initiated for the treatment of chronic pain. This, despite the growing evidence showing a lack of effectivity and an increased risk of complications such as the development of substance use disorders. Specifically, people with MH problems appear to be more vulnerable to lose control and develop opioid use disorders.

**Disclosure of Interest:**

None Declared

